# Digenic inheritance of *MSH6* and *MUTYH* variants in familial colorectal cancer

**DOI:** 10.1002/gcc.22883

**Published:** 2020-08-11

**Authors:** Stephanie A. Schubert, Dina Ruano, Yvonne Tiersma, Mark Drost, Niels de Wind, Maartje Nielsen, Liselotte P. van Hest, Hans Morreau, Noel F. C. C. de Miranda, Tom van Wezel

**Affiliations:** ^1^ Department of Pathology Leiden University Medical Center Leiden The Netherlands; ^2^ Department of Human Genetics Leiden University Medical Center Leiden The Netherlands; ^3^ Department of Clinical Genetics Leiden University Medical Center Leiden The Netherlands; ^4^ Department of Clinical Genetics Amsterdam UMC, Vrije Universiteit Amsterdam Amsterdam The Netherlands

**Keywords:** digenic inheritance, familial colorectal cancer, Lynch syndrome, MSH6, MUTYH, whole‐exome sequencing

## Abstract

We describe a family severely affected by colorectal cancer (CRC) where whole‐exome sequencing identified the coinheritance of the germline variants encoding MSH6 p.Thr1100Met and MUTYH p.Tyr179Cys in, at least, three CRC patients diagnosed before 60 years of age. Digenic inheritance of monoallelic *MSH6* variants of uncertain significance and *MUTYH* variants has been suggested to predispose to Lynch syndrome‐associated cancers; however, cosegregation with disease in the familial setting has not yet been established. The identification of individuals carrying multiple potential cancer risk variants is expected to rise with the increased application of whole‐genome sequencing and large multigene panel testing in clinical genetic counseling of familial cancer patients. Here we demonstrate the coinheritance of monoallelic variants in *MSH6* and *MUTYH* consistent with cosegregation with CRC, further supporting a role for digenic inheritance in cancer predisposition.

## 1 INTRODUCTION

Approximately 25% of colorectal cancers (CRCs) are diagnosed in patients with a family history of CRC. However, the majority of familial CRC cannot be explained by clear‐cut genetic defects, which hampers appropriate genetic counselling.^1^ The most frequent form of hereditary CRC is Lynch syndrome (OMIM#120435), which predisposes to cancers that develop in a context of DNA mismatch repair (MMR) deficiency, including CRC and endometrial cancer. It is caused by heterozygous, pathogenic variants affecting the DNA MMR genes, *MLH1*, *MSH2*, *MSH6*, or *PMS2*. MUTYH‐associated polyposis (MAP; OMIM#608456) is a recessively inherited CRC syndrome caused by biallelic variants in the base‐excision repair gene *MUTYH*. The potential of monoallelic, pathogenic *MUTYH* variants to predispose to CRC remains debatable.^1^ Some *MUTYH* variants confer greater functional defects in vitro and are associated with more severe clinical phenotypes, such as the variant encoding p.Tyr179Cys compared to p.Gly396Asp.^2^, ^3^

Digenic inheritance of monoallelic *MSH6* and *MUTYH* variants has been suggested to predispose to Lynch syndrome‐associated cancers; however, cosegregation of both variants within CRC families has not yet been demonstrated.^4^, ^5^, ^6^, ^7^, ^8^, ^9^ Here, we demonstrate, for the first time, the coinheritance of monoallelic variants in *MSH6* and *MUTYH* consistent with the cosegregation with CRC, further supporting a role for digenic inheritance in cancer predisposition.

## 2 MATERIALS AND METHODS

### 2.1 Patients

Clinicopathological data of family members was obtained during consultations at the department of Clinical Genetics of the Amsterdam University Medical Centre, Vrije Universiteit Amsterdam. DNA was extracted from peripheral blood and formalin‐fixed paraffin‐imbedded tissues using standard techniques. All patients provided written informed consent. The study was approved by the Medical Ethical Committee of the Leiden University Medical Center, The Netherlands (protocol P01.019).

### 2.2 Whole‐exome sequencing

Whole‐exome sequencing was outsourced to BGI (BGI‐Shenzhen, Shenzhen, China); exome libraries were constructed with the BGI capture kit, followed by sequencing on the Complete Genomics' Sequencing Platform (Complete Genomics Inc., San Jose, California). Filtering and variant prioritization was performed as previously described.^10^ All variants were selected based on a maximum population frequency <0.01 (in 1000 Genomes phase 3, ExAC 1.0, ESP6500SI‐V2 or GoNL release 5).

### 2.3 Variant screening

The *MSH6* (p.Thr1100Met) and *MUTYH* (p.Tyr179Cys) variants were validated and investigated in additional family members by using Sanger sequencing of PCR products obtained under standard PCR conditions. The following M13‐tailed primer sets were used: 5′‐TGT AAA ACG ACG GCC AGT AAA ACC CCC AAA CGA TGA A‐3′ and 5′‐CAG GAA ACA GCT ATG ACC TGC TCC TCT TCC TCA CAG‐3′ for *MSH6*, and 5′‐GAC GTT GTA AAA CGA CGG CCA GTC CCT AGG GTA GGG GAA ATA GG‐3′ and 5′‐CAG GAA ACA GCT ATG ACC ATG AGT TCC TAC CCT CCT GCC ATC‐3′ for *MUTYH* (M13‐tails are underlined).

### 2.4 Tumor analysis

MMR deficiency in tumor samples was assessed by microsatellite instability analysis and immunohistochemical detection of the four MMR proteins (MLH1, MSH2, MSH6, and PMS2).^11^ *KRAS* codon 12/13 mutations were screened with Sanger sequencing.^12^

### 2.5 Functional MMR assay

In vitro MMR activity assay was performed as previously described.^13^

## 3 RESULTS

**FIGURE 1:**
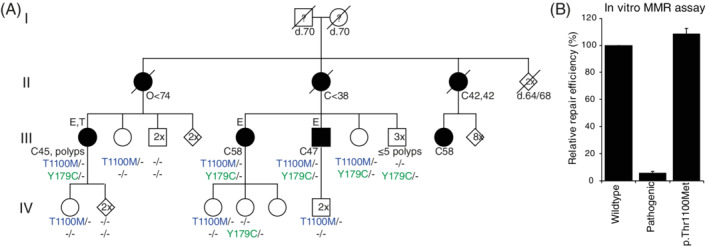
The digenic inheritance of *MSH6* and *MUTYH* variants. A, The pedigree shows the coinheritance of the monoallelic variants which encode MSH6 p.Thr1100Met and MUTYH p.Tyr179Cys in a family affected by colorectal cancer. All spouses were unrelated and unaffected by cancer. Genotypes: *MSH6* p.Thr1100Met (T1100M; blue); *MUTYH* p.Tyr179Cys (Y179C; green); ‐, wild type. E, whole‐exome sequencing analysis; T, tumor analysis; ?, unknown phenotype; numbers in symbols, number of unaffected relatives merged for clarity; filled symbols, cancer patients; C, colorectal cancer; E, endometrial cancer; O, ovarian cancer; d., age at death; followed by the age at diagnosis or death. B, in vitro mismatch repair (MMR) activity assay shows wild‐type MMR activity of MSH6 p.Thr1100Met, compared to wild‐type MSH6 (p.Gly529Gly) and a pathogenic MSH6 mutant (p.Gly1139Ser). Data are shown as mean ± SEM of three independent experiments [Color figure can be viewed at wileyonlinelibrary.com]

We performed germline whole‐exome sequencing on three CRC patients diagnosed before 60 years of age (III‐1, III‐7, III‐8, Figure 1A) and who belonged to a CRC family comprising of seven cancer patients divided over two generations. Twenty‐two rare variants were shared by the three patients (Tables 1 and S1), including variants in the *MSH6* (NM_000179.2: c.3299C > T, p.Thr1100Met) and *MUTYH* (NM_001128425.1: c.536A > G, p.Tyr179Cys) genes, while the other 20 genes could not be clearly linked to cancer predisposition. The identified *MSH6* variant was classified as a variant of uncertain significance (VUS) in the Leiden Open Variant Database and the InSiGHT DNA Variant Database.^14^, ^15^The *MUTYH* variant is the most common pathogenic variant found in the Netherlands.^2^

**TABLE 1:** All rare variants shared by the three individuals from whole‐exome sequencing data

Chr	Gene	RefSeq accession number	mRNA change	Protein change	Population frequency^a^	ClinVar classification^b^	Franklin classification^c^	Cancer gene census
1	*EBNA1BP2*	NM_001159936	c.1034A > T	p.Asn345Ile	0.006009	—	Benign	—
**1**	***MUTYH***	**NM_001128425**	**c.536A > G**	**p.Tyr179Cys**	**0.001538**	**Pathogenic**	**Pathogenic**	**Yes**
1	*TESK2*	NM_007170	c.983A > G	p.Gln328Arg	0.0006052	—	VUS	—
1	*CAPN9*	NM_006615	c.55G > T	p.Ala19Ser	0.00006365	—	VUS	—
**2**	***MSH6***	**NM_000179**	**c.3299C > T**	**p.Thr1100Met**	**0.00004243**	**Uncertain**	**VUS**	**Yes**
3	*C3orf20*	NM_032137	c.1746C > G	p.Phe582Leu	0.005847	—	Likely benign	—
5	*DNAH5*	NM_001369	c.1781A > G	p.Glu594Gly	—	—	VUS	—
7	*KIAA1324L*	NM_001142749	c.2369 T > C	p.Val790Ala	0.0006585	—	VUS	—
7	*TRIP6*	NM_003302	c.822G > C	p.Glu274Asp	0.0009893	—	VUS	—
7	*CUX1*	NM_001202543	c.1438A > G	p.Ser480Gly	0.001128	—	Likely benign	Yes
7	*ZNF783*	NM_001195220	c.46A > G	p.Thr16Ala	0.001083	—	VUS	—
8	*PDP1*	NM_018444	c.283A > C	p.Ser95Arg	—	—	VUS	—
9	*NMRK1*	NM_017881	c.304C > G	p.Leu102Val	0.001419	—	VUS	—
9	*GAPVD1*	NM_015635	c.850G > A	p.Val284Met	0.003596	—	Benign	—
11	*INTS5*	NM_030628	c.1436A > G	p.Asn479Ser	0.00004607	—	VUS	—
11	*GAL3ST3*	NM_033036	c.326G > A	p.Arg109His	0.00004731	—	VUS	—
11	*SORL1*	NM_003105	c.3346A > G	p.Ile1116Val	0.005308	—	VUS	—
14	*LTBP2*	NM_000428	c.1226G > A	p.Arg409His	0.0000203	—	VUS	—
15	*RYR3*	NM_001036	c.7812C > G	p.Asn2604Lys	0.002144	Likely benign	Likely benign	—
15	*DAPK2*	NM_014326	c.179G > A	p.Arg60Gln	0.003725	—	Likely benign	—
16	*NLRC5*	NM_032206	c.1219G > A	p.Ala407Thr	0.000003542	—	VUS	—
20	*C20orf85*	NM_178456	c.101G > A	p.Arg34Gln	0.00192	—	Likely benign	—

Abbreviations: Chr, chromosome; VUS, variant of uncertain significance.

^a^ Population frequency (gnomAD 2.1.1).

^b^ ClinVar clinical significance (ClinVar database version August 5, 2019).

^c^ Franklin by Genoox (accessed on May 20, 2020).

Fourteen relatives, all unaffected by cancer or polyposis, were genotyped for these *MSH6* and *MUTYH* variants, identifying one additional carrier of both variants, five *MSH6*‐only carriers and four *MUTYH*‐only carriers. In all probability, the mothers of the sequenced patients, II‐1 and II‐2, who were affected by ovarian cancer bellow age 74 and CRC at 38 years old respectively, were obligate carriers of both variants; however, DNA was unavailable for testing and, formally, inheritance through the fathers to the sequenced individuals (III‐1, III‐7, III‐8) cannot be excluded. MMR deficiency was not detected in the colorectal carcinoma of patient III‐1, which also lacked the *KRAS* mutation typical for MAP tumors (c.34C > T; Table S2). Functional analysis of the MSH6 p.Thr1100Met variant showed retained MMR function in vitro (Figure 1B).

## 4 DISCUSSION

Digenic inheritance of monoallelic *MSH6* and *MUTYH* variants has been suggested to predispose to Lynch syndrome‐associated cancers. The involvement of both MSH6 and MUTYH in oxidative DNA damage repair and their physical interaction enhancing MUTYH's repair activity, substantiates the association of variants in these genes.^16^ From earlier studies, the inheritance of monoallelic *MUTYH* variants seemed primarily relevant in patients carrying *MSH6* VUSs, which are less strongly associated with MMR deficiency than pathogenic *MSH6* variants (Table S2).^4^, ^5^, ^6^, ^7^, ^8^, ^9^ Furthermore, a digenic inheritance model was proposed once before for CRC predisposition in a carrier of variants in the oxidative DNA damage repair genes *MUTYH* and *OGG1*.^17^ Although the functional evidence of combined defects in oxidative DNA damage repair genes is still lacking, the coinheritance of *MSH6* and *MUTYH* variants in at least three, but likely five cancer cases within one family warrants further mechanistic and clinical studies. The absence of cancer and numerous polyps in nondigenic carriers further substantiates this association. Tumor analysis of the tumor of one of the digenic carriers and the in vitro MMR activity assay indicated retention of MMR function of MSH6 p.Thr1100Met protein. In addition, the genetic marker for MAP‐tumors (*KRAS* c.34G > T) was absent in this tumor, which points toward retained MUTYH repair activity. The combined inheritance of both genetic variants could still result in impaired repair of oxidative DNA damage. More extensive somatic mutation analysis to assess this was, however, not possible, because of low quality of the DNA sample and the unavailability of additional tumor material.

Next to *MSH6* and *MUTYH*, *CUX1* has been described as a cancer‐driving gene.^18^ *CUX1* is implicated in inflammatory bowel disease and various cancer types, although primarily due to loss‐of‐function somatic mutations.^18^, ^19^ This gene codes for several isoforms, including the ubiquitously expressed p200 CUX1, which, among other functions, has been shown to stimulate the repair of oxidized DNA bases by OGG1.^20^ The identified *CUX1* (NM_001202543: c.1438A > G, p.Ser480Gly) variant, however, was classified as likely benign by the Franklin variant classification tool.^21^ Additional gene reportedly linked to tumorigenesis include *RYR3*,^22^ *EBNA1BP2*,^23^ *TRIP6*,^24^ and *CAPN9*.^25^ The *RYR3* (NM_001036: c.7812C > G, p.Asn2604Lys) and *EBNA1BP2* (NM_001159936: c.1034A > T, p.Asn345Ile) variants were classified as likely benign and benign, respectively, while the *TRIP6* (NM_003302: c.822G > C, p.Glu274Asp) and the *CAPN9* (NM_006615: c.55G > T, p.Ala19Ser) variants were classified as VUS.^21^ TRIP6 promotes cell migration and invasion through Wnt/β‐catenin signaling and was shown to be upregulated in colorectal tumors.^24^ Therefore, *TRIP6* variants that increase protein stability or expression could potentially stimulate colorectal tumorigenesis. In addition, lost‐of‐function variants in *CAPN9* might promote tumor formation, as Calpain‐9 induces cell cycle arrest and apoptosis, and low expression predicts a poorer prognosis in gastric cancer patients.^25^ The contribution of the genetic variants, other than *MSH6* and *MUTYH*, to cancer risk cannot be completely excluded. However, none of these variants have been functionally investigated and especially the variants predicted as benign or likely benign are less likely to contribute to an increased cancer risk. Besides, none of these genes have, to date, been associated with a genetic predisposition to any types of cancer.

In conclusion, with the increased application of whole‐genome sequencing or large multigene panel testing in clinical genetic counseling, the number of identified individuals carrying multiple potential risk variants is expected to rise. Here, we demonstrate the coinheritance of *MSH6* and *MUTYH* variants consistent with the cosegregation with cancer, further supporting a role for digenic inheritance in CRC predisposition. Our results reiterate that digenic inheritance should be considered as cause of genetic diseases.

## CONFLICT OF INTEREST

The authors declare no conflicts of interest.

## AUTHOR CONTRIBUTIONS

Tom van Wezel, Noel F. C. C. de Miranda, and Hans Morreau conceived and designed the study. Dina Ruano performed next‐generation sequencing analyses. Noel F. C. C. de Miranda and Stephanie A. Schubert performed analysis and interpretation of whole‐exome sequencing data. Mark Drost and Yvonne Tiersma performed functional analysis. Maartje Nielsen and Liselotte P. van Hest performed patient counseling and clinical data acquisition. Hans Morreau performed the pathology review of the samples. Tom van Wezel, Noel F. C. C. de Miranda, Mark Drost, and Niels de Wind supervised the work. Stephanie A. Schubert, Noel F. C. C. de Miranda, and Tom van Wezel wrote the manuscript. All authors read and approved the manuscript.

## Supporting information


**Supplementary Table S1** . All rare variants shared by the three individuals from whole‐exome sequencing data
**Supplementary Table S2**: Digenic inheritance of MSH6 and MUTYH variants.

## Data Availability

The data that support the findings of this study are available from the corresponding author upon reasonable request. The data are not publicly available due to privacy restrictions.
